# Real-Time Measurement and Uncertainty Evaluation of Optical Path Difference in Fiber Optic Interferometer Based on Auxiliary Interferometer

**DOI:** 10.3390/s24072038

**Published:** 2024-03-22

**Authors:** Huicong Li, Minggan Lou, Wenzhu Huang, Wentao Zhang

**Affiliations:** 1 State Key Laboratory of Transducer Technology, Institute of Semiconductors, Chinese Academy of Sciences, Beijing 100083, China; hcli@semi.ac.cn (H.L.); mglou@semi.ac.cn (M.L.); hwzhu@semi.ac.cn (W.H.); 2 Center of Materials Science and Optoelectronic Engineering, University of Chinese Academy of Sciences, Beijing 100049, China; 3 Shenzhen Academy of Disaster Prevention and Reduction, Shenzhen 518003, China

**Keywords:** optical path difference, real-time measurement, measurement uncertainty, auxiliary interferometer

## Abstract

Optical interferometers are the main elements of interferometric sensing and measurement systems. Measuring their optical path difference (OPD) in real time and evaluating the measurement uncertainty are key to optimizing system noise and ensuring system consistency. With the continuous sinusoidal wavelength modulation of the laser, real-time OPD measurement of the main interferometer is achieved through phase comparison of the main and auxiliary interferometers. The measurement uncertainty of the main interferometer OPD is evaluated. It is the first evaluation of the impact of different auxiliary interferometer calibration methods on OPD measurements. A homodyne quadrature laser interferometer (HQLI) is used as the main interferometer, and a 3 × 3 interferometer is used as the auxiliary interferometer. The calibration of the auxiliary interferometer using optical spectrum analyzer scanning and ruler measurement is compared. The evaluation shows that the auxiliary interferometer is the most significant source of uncertainty and causes the total uncertainty to increase linearly with increasing OPD. It is proven that a high-precision calibration and large-OPD auxiliary interferometer can improve the real-time accuracy of OPD measurements based on the auxiliary interferometer. The scheme can determine the minimum uncertainty to optimize the system noise and consistency for vibration, hydroacoustic, and magnetic field measurements with OPDs of the ~m level.

## 1. Introduction

Optical interferometers transmit signals based on the interference of the superimposed waves, with the advantages of high resolution, high sensitivity, and wide dynamic range. Typical examples are the Michelson interferometer, Mach–Zehnder interferometer, Fabry–Pérot interferometer, and Sagnac interferometer. As the main elements, they are widely used in pointwise sensing or measurement, such as displacement [1,2], vibration [3], hydrophone [4], and other fields. They also can be used in temperature [5], strain [6], refractive index [7], and other distributed sensing and measurement fields as auxiliary elements.

The performance of optical interferometers is closely related to their optical path difference (OPD) or arm length difference [8]. For unbalanced interferometers, the OPD introduces laser frequency noise [8,9], and selecting an appropriate OPD is essential for system performance [10]. For balanced interferometers, achieving zero OPD helps obtain a lower system noise level [11]. Therefore, determining the interferometer OPD is crucial and meaningful for the performance optimization of the interferometric system.

In recent decades, many schemes have been developed to measure interferometer OPD. Some schemes are even used for absolute distance measurements or long-distance ranging. Still, they can also be used in OPD measurements, such as white-light interferometry (WLI) [12,13,14], frequency scanning interferometry (FSI) [15,16], and frequency-modulated continuous-wave (FMCW) technology [17,18]. WLI uses white light or broadband light sources to interrogate the optical interferometer and measure the OPD through optical path matching [19], optical spectrum analyzer (OSA) scanning [13,20], or wavelength scanning [14], which has high measurement accuracy. However, optical path matching requires high-precision mechanical scanning devices, OSA scanning requires waiting for the response time, and wavelength scanning requires additional wavelength measurement devices for monitoring. These schemes do not have the real-time performance of OPD measurements. Compared with WLI, FSI uses a linear sweeping laser to sweep a wide range of frequencies (up to several hundred GHz) and then solves the proportional relationship between the optical frequency change and the phase change; that is, an absolute measurement of the interferometer OPD can be achieved. Gas absorption spectroscopy, optical frequency comb (OFC), and the Fabry-Pérot interferometer [21] can be used to monitor the optical frequency changes of FSI. However, their discrete spectra cannot monitor the entire frequency scanning process in real time, and the OPD is challenging to measure accurately in real time. For FMCW technology, calculating the OPD from a constant beat frequency is not sufficient in real time [17,22]. The auxiliary interferometer is a simple and effective scheme to ensure the real-time measurement of OPD [16,18,23]. The OPD measurement made by FSI or FMCW technology is based on the auxiliary interferometer, which serves as the benchmark and significantly impacts OPD measurement accuracy. In short, the influence of the auxiliary interferometer calibration method on the OPD measurement of the main interferometer under test should be considered to obtain a more accurate OPD measurement. However, the current FSI and FMCW technology based on the auxiliary interferometer rarely analyze the uncertainty contributed by the known OPD calibration method of the auxiliary interferometer [16].

In this paper, we use a calibrated auxiliary interferometer and laser wavelength modulation for real-time OPD measurement of the main interferometer. We focus on evaluating the measurement uncertainty caused by phase comparison and auxiliary interferometer calibration methods and analyze the impact of different auxiliary interferometer calibration methods on real-time OPD measurements. The proposed scheme and uncertainty evaluation are applied to a homodyne quadrature laser interferometer using a 3 × 3 interferometer as an auxiliary. The experimental results show a real-time OPD measurement range of 0~20 mm, and the magnitudes of the uncertainties within the measurement range are evaluated. It is determined that the auxiliary interferometer is the main source of measurement uncertainty, which provides reference guidance for optimizing the interferometer system.

## 2. Measurement Principle

### 2.1. Optical Path Difference Measurement Scheme

**Figure 1:**
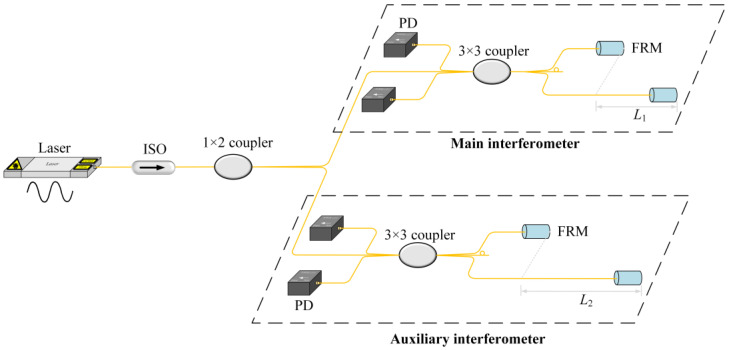
Real-time measurement principle of main interferometer’s OPD. ISO, isolator; PD, photodetector; FRM, Faraday rotator mirror.

Due to the auxiliary interferometer with a known OPD, the main interferometer with an unknown OPD will be measured through phase comparison. As shown in Figure 1, the main interferometer and the auxiliary interferometer are Michelson interferometers, both interrogated by the same laser. By fast tuning the wavelength of the laser (tuning range of several pm), such as sinusoidal modulation, phase changes of two interferometers are produced. The greater the amplitude of the phase change, the greater the corresponding OPD. Here, the phase changes can be measured simultaneously because the wavelength modulations of the main interferometer and the auxiliary interferometer are consistent. Namely, the OPD measurement is in real time. Moreover, using the auxiliary interferometer can eliminate the error that may be introduced by laser instability.

The unknown OPD of the main interferometer is *L*_1_, and the known OPD of the auxiliary interferometer is *L*_2_. By fast-tuning the laser wavelength, the phase change of the main interferometer and that of the auxiliary interferometer are, respectively,

(1) $$\Delta \varphi_{1}=\frac{4\pi }{\lambda^{2}}\Delta \lambda L_{1},$$

(2) $$\Delta \varphi_{2}=\frac{4\pi }{\lambda^{2}}\Delta \lambda L_{2},$$

where *λ* is the center wavelength of the laser, and Δ*λ* is the wavelength change of the laser.

The phase change of each interferometer is proportional to its OPD. The OPD of the main interferometer can be measured by comparing the phase changes. This kind of phase comparison uses the same fast-tuning laser. It does not need to know the tuning amplitude and frequency of the laser wavelength, thus avoiding the interference of the laser on the measurement as much as possible. The OPD of the main interferometer is calculated as follows:

(3) $$L_{1}=\frac{\Delta \varphi_{1}}{\Delta \varphi_{2}}L_{2}.$$

By calibrating the OPD of the auxiliary interferometer in advance, the OPD of the main interferometer can be obtained in real time only by conducting real-time calculations of the phase comparison of the two interferometers. In general, the auxiliary interferometer is all-fiber. Due to the variability in the optical fiber in the preform production process, the refractive index of the optical fiber is not necessarily a constant value. The auxiliary interferometer is interrogated with white light, and its spectrum is measured through an OSA [20]. If the wavelengths corresponding to adjacent valley powers are *λ*_1_ and *λ*_2,_ respectively, then the OPD of the auxiliary interferometer *L*_2_ is

(4) $$L_{2}=\frac{\lambda_{1}\lambda_{2}}{2\left(\lambda_{1}-\lambda_{2}\right)}.$$

Then, based on the auxiliary interferometer calibrated by OSA scanning, the measurement formula of the OPD of the main interferometer is

(5) $$L_{1}=\frac{\Delta \varphi_{1}}{\Delta \varphi_{2}}\cdot \frac{\lambda_{1}\lambda_{2}}{2\left(\lambda_{1}-\lambda_{2}\right)}.$$

### 2.2. Phase Demodulation

The main interferometer and the auxiliary interferometer can construct two or more detection signals through a 3 × 3 coupler or other methods. There is a stable phase angle between the multi-detection signals. For example, the interferometer comprises a 3 × 3 coupler and two Faraday rotator mirrors (FRMs). Any two output optical signals in the 3 × 3 coupler have an ideal phase angle of 120°. Here, the ellipse fitting algorithm (EFA) based on the Kalman filter [24] is used to simultaneously demodulate the phase changes Δ*φ*_1_ and Δ*φ*_2_ of the main and auxiliary interferometers, respectively. The simultaneous demodulation avoids the errors caused by time differences and algorithm differences.

The EFA based on the Kalman filter includes two stages: ellipse fitting and Kalman filter correction. In our previous work, we introduced the application of Kalman filter correction in the EFA in detail [24]. Kalman filter correction ensures the real-time updating of interference fringe parameters to reduce errors caused by inherent defects of the 3 × 3 coupler and light-intensity fluctuations. Here, we mainly introduce the phase demodulation process of ellipse fitting.

The interference signals from the optical interferometer detected by the photodetectors (PDs) are [24]

(6) $$\begin{array}{l} V_{x}=V_{x0}+V_{x1}\cos\left(\Delta \varphi +\delta_{xy}-\pi /2\right)\\ V_{y}=V_{y0}+V_{y1}\sin\left(\Delta \varphi \right) \end{array},$$

where *V_x_*_0_ and *V_y_*_0_ are DC offsets of the interference signals; *V_x_*_1_ and *V_y_*_1_ are AC amplitudes of the interference signals; *δ_xy_* is the phase angle between the two interference signals.

The phase change Δ*φ* of the interferometer can be calculated by

(7) $$\Delta \varphi =\arctan\left[\frac{V_{y}-V_{y0}}{\xi_{xy}\left(V_{x}-V_{x0}\right)+\gamma_{xy}\left(V_{y}-V_{y0}\right)}\right],$$

where *ξ_xy_* and *γ_xy_* are defined, respectively, as

(8) $$\xi_{xy}=\frac{V_{y1}/V_{x1}}{\sin\delta_{xy}},\;\gamma_{xy}=\tan\left(\delta_{xy}-\pi /2\right).$$

The EFA is developed based on Heydeman correction [25], and its essence is to fit the best curve parameters by expressing Equation (6) as a general form of a conic section. The conic section is

(9) $$AV_{x}^{2}+BV_{x}V_{y}+CV_{y}^{2}+DV_{x}+EV_{y}+F=0.$$

Here, the curve parameters (*A*, *B*, *C*, *D*, *E*, and *F*) are calculated based on the least squares method, and then (*V_x_*_0_, *V_y_*_0_, *ξ_xy_*, and *γ_xy_*) are solved with the following expressions:

(10) $$\begin{array}{c} V_{x0}=\frac{BE-2CD}{4AC-B^{2}}\\ V_{y0}=\frac{BD-2AE}{4AC-B^{2}}\\ \xi_{xy}=\frac{2A}{\sqrt{4AC-B^{2}}}\\ \gamma_{xy}=\frac{B}{\sqrt{4AC-B^{2}}} \end{array}.$$

The phase changes of the interferometers can be calculated by substituting (*V_x_*_0_, *V_y_*_0_, *ξ_xy_*, and *γ_xy_*) into Equation (7). Therefore, the main and auxiliary interferometers should be able to calculate their respective phase changes under laser wavelength tuning using the above algorithm.

### 2.3. Measurement Uncertainty Evaluation

According to Equation (5), the OPD measurement of the main interferometer is related to the phase comparison of the two interferometers and the calibration method of the auxiliary interferometer.

The uncertainties introduced by phase comparison include the component introduced by the measurement repeatability of the phase change Δ*φ*_1_ (*u*_1_), the component introduced by the measurement repeatability of the phase change Δ*φ*_2_ (*u*_2_), and the component introduced by phase demodulation accuracy (*u*_3_), which can be expressed as

(11) $$\begin{array}{ll} u_{p} & =\sqrt{u_{1}^{2}+u_{2}^{2}+u_{3}^{2}}\\ & =\sqrt{{\left[\frac{1}{\Delta \varphi_{2}}\cdot \frac{\lambda_{1}\lambda_{2}}{2\left(\lambda_{1}-\lambda_{2}\right)}\right]}^{2}\sigma_{\Delta \varphi_{1}}^{2}+{\left[-\frac{\Delta \varphi_{1}}{{\Delta \varphi_{2}}^{2}}\cdot \frac{\lambda_{1}\lambda_{2}}{2\left(\lambda_{1}-\lambda_{2}\right)}\right]}^{2}\sigma_{\Delta \varphi_{2}}^{2}+\left\{{\left[\frac{1}{\Delta \varphi_{2}}\cdot \frac{\lambda_{1}\lambda_{2}}{2\left(\lambda_{1}-\lambda_{2}\right)}\right]}^{2}+{\left[-\frac{\Delta \varphi_{1}}{{\Delta \varphi_{2}}^{2}}\cdot \frac{\lambda_{1}\lambda_{2}}{2\left(\lambda_{1}-\lambda_{2}\right)}\right]}^{2}\right\}\sigma_{p}^{2}} \end{array},$$

where *σ*_Δ*φ*1_ is the standard deviation of the average value of repeated measurements of Δ*φ*_1_, *σ*_Δ*φ*2_ is the standard deviation of the average value of repeated measurements of Δ*φ*_2_, and *σ_p_* is the phase demodulation accuracy, presented as the standard deviation.

The calibration method of the auxiliary interferometer is OSA scanning. Thus, the uncertainties introduced by OSA scanning include the component introduced by the measurement repeatability of trough *λ*_1_ (*u*_4_), the component introduced by the measurement repeatability of trough *λ*_2_ (*u*_5_), and the component introduced by the resolution of the OSA (*u*_6_). The resolution of the OSA is *r*, and its corresponding uncertainty is *u_r_* = *r*/2. Therefore, the uncertainty caused by wavelength reading is

(12) $$\begin{array}{ll} u_{\mathrm{OSA}} & =\sqrt{u_{4}^{2}+u_{5}^{2}+u_{6}^{2}}\\ & =\sqrt{{\left[-\frac{\Delta \varphi_{1}}{\Delta \varphi_{2}}\cdot \frac{{\lambda_{2}}^{2}}{2{\left(\lambda_{1}-\lambda_{2}\right)}^{2}}\right]}^{2}\sigma_{\lambda_{1}}^{2}+{\left[\frac{\Delta \varphi_{1}}{\Delta \varphi_{2}}\cdot \frac{{\lambda_{1}}^{2}}{2{\left(\lambda_{1}-\lambda_{2}\right)}^{2}}\right]}^{2}\sigma_{\lambda_{2}}^{2}+\left\{{\left[-\frac{\Delta \varphi_{1}}{\Delta \varphi_{2}}\cdot \frac{{\lambda_{2}}^{2}}{2{\left(\lambda_{1}-\lambda_{2}\right)}^{2}}\right]}^{2}+{\left[\frac{\Delta \varphi_{1}}{\Delta \varphi_{2}}\cdot \frac{{\lambda_{1}}^{2}}{2{\left(\lambda_{1}-\lambda_{2}\right)}^{2}}\right]}^{2}\right\}\sigma_{r}^{2}} \end{array},$$

where *σ_λ_*_1_ is the standard deviation of the average value of repeated measurements of *λ*_1_, and *σ_λ_*_2_ is the standard deviation of the average value of repeated measurements of *λ*_2_.

In summary, the OPD measurement uncertainty of the main interferometer is evaluated as $u=\sqrt{u_{p}^{2}+u_{\mathrm{OSA}}^{2}}$. Furthermore, for different values of the OPD *L*_1_ of the main interferometer, the expression of the relative uncertainty is

(13) $$\begin{array}{ll} \frac{u}{L_{1}} & =\sqrt{{\left(\frac{u_{1}}{L_{1}}\right)}^{2}+{\left(\frac{u_{2}}{L_{1}}\right)}^{2}+{\left(\frac{u_{3}}{L_{1}}\right)}^{2}+{\left(\frac{u_{4}}{L_{1}}\right)}^{2}+{\left(\frac{u_{5}}{L_{1}}\right)}^{2}+{\left(\frac{u_{6}}{L_{1}}\right)}^{2}}\\ & =\sqrt{\begin{array}{l} {\left(\frac{\sigma_{\Delta \varphi_{1}}}{\Delta \varphi_{1}}\right)}^{2}+{\left(\frac{\sigma_{\Delta \varphi_{2}}}{\Delta \varphi_{2}}\right)}^{2}+\left[{\left(\frac{1}{\Delta \varphi_{1}}\right)}^{2}+{\left(\frac{1}{\Delta \varphi_{2}}\right)}^{2}\right]{\sigma_{p}}^{2}+{\left(\frac{\lambda_{2}}{\lambda_{1}\left(\lambda_{1}-\lambda_{2}\right)}\sigma_{\lambda_{1}}\right)}^{2}+{\left(\frac{\lambda_{1}}{\lambda_{2}\left(\lambda_{1}-\lambda_{2}\right)}\sigma_{\lambda_{2}}\right)}^{2}\\ +\left[\frac{{\lambda_{2}}^{2}}{{\lambda_{1}}^{2}{\left(\lambda_{1}-\lambda_{2}\right)}^{2}}+\frac{{\lambda_{1}}^{2}}{{\lambda_{2}}^{2}{\left(\lambda_{1}-\lambda_{2}\right)}^{2}}\right]{u_{r}}^{2} \end{array}} \end{array}.$$

This expression means that when measuring the unknown OPD of the main interferometer, if the OPD increases and the relative uncertainty components *u*_2_/*L*_1_, *u*_4_/*L*_1_, *u*_5_/*L*_1_, and *u*_6_/*L*_1_ are considered unchanged, an increase in the phase change Δ*φ*_1_ of the main interferometer can be expected to lead to an overall reduction in the relative uncertainty, which is very meaningful for long-distance ranging. We will show this correlation again in subsequent experiments. Some similar schemes [18,26] have long been used in long-distance ranging.

## 3. Experimental Setups

### 3.1. Calibration of the Auxiliary Interferometer

**Figure 2:**
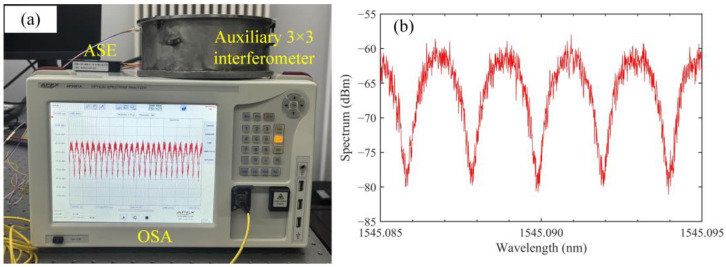
Experimental photo (**a**) and interferometric spectrum (**b**) of auxiliary 3 × 3 interferometer calibration.

A 3 × 3 coupler and two FRMs are fused into a fiber optic unbalanced Michelson interferometer as an auxiliary. The arm length difference of the auxiliary 3 × 3 interferometer is about 40 cm, which can ensure better measurement accuracy. In this way, there can be a phase change greater than 2π under laser wavelength modulation to correct the influence of interference light-intensity fluctuations in real time through the Kalman filter, and it can also avoid the noise of the enhancement of fiber Rayleigh scattering due to the longer arm length [27]. The auxiliary 3 × 3 interferometer is well protected in sound, vibration, and temperature isolation insulation, which aims to minimize the subtle changes in the OPD of the interferometer caused by external factors, as shown in Figure 2a.

White light with a spectrum range of 38 nm is provided by an amplified spontaneous emission (ASE) light source (Shenzhen Hoyatek, Shenzhen, China, HY-ASE-C-13-G-M-FA-CIR), and the interference spectrum of the 3 × 3 interferometer is measured by an OSA (APEX, Technologies, Marcoussis, France, AP2061A), as shown in Figure 2a. The resolution of the OSA is set to 0.04 pm. Ten measurements are taken. The measurement process must not be in contact with the optical table and must be kept quiet to avoid disturbing the OPD of the auxiliary 3 × 3 interferometer. Figure 2b shows the auxiliary interferometer’s interference spectrum. Taking the *λ*_1_ and *λ*_2_ values that are close to each other and averaging them, *λ*_1_ = 1545.08813 nm and *λ*_2_ = 1545.08609 nm (the number of decimal points are truncated at the resolution level of the OSA). Calculated by Equation (5), the OPD of the auxiliary 3 × 3 interferometer is approximately 0.583405 m.

### 3.2. Setup of OPD Measurement

**Figure 3:**
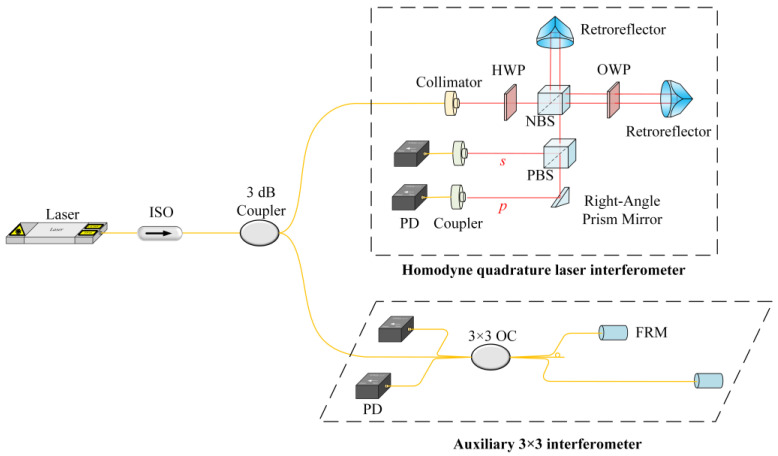
OPD measurement experiment of HQLI.

The main interferometer is a homodyne quadrature laser interferometer (HQLI), and the measurement optical path is set up according to Figure 3. Each component in the HQLI is fixed on a stable optical tabletop (Newport, Irvine, CA, USA, M-ST-UT2-510-8) through rigid connections. Its OPD measurement is performed in a super clean room with a stable environment. Both interferometers are interrogated by a laser (NKT Photonics, Birkerød, Denmark, Koheras BASIK E15), which has a wavelength of 1537.35 nm. Before employing the collimator (Oz optics, Carp, ON, Canada, HPUC-23AF-1300/1550-S-6.2AS-11) of the HQLI, a polarization control analyzer (General Photonics, Chino, CA, USA, PSY-201) is used to stabilize the laser into linearly polarized light whose polarization direction is perpendicular to the optical tabletop. This setup is not shown in Figure 3, but it is an unavoidable operation.

For the HQLI, the incident linearly polarized light is rotated by a half-wave plate (HWP) (Thorlabs, Newton, NJ, USA, WPH05ME-1550), and the polarization direction of the laser is 45° in the vertical direction of the optical tabletop so that it passes through a nonpolarizing beam splitter (NBS) (Thorlabs, Newton, NJ, USA, BS012). The amplitudes are equal in the *p* and *s* polarization components. After the laser passes through the NBS, it is divided into two beams in a 50/50 split. The beam reflected by the NBS passes through the retroreflector (Thorlabs, Newton, NJ, USA, PS974-C) to achieve 180° refraction of the optical path; the beam transmitted by the NBS passes through the octadic-wave plate (OWP) (Thorlabs, Newton, NJ, USA, PLCC0178) and is reflected by the retroreflector. The fast axis of the OWP is perpendicular to the optical tabletop. The laser passes through the OWP twice, equivalent to passing through a quarter-wave plate once. The phase of one polarization component relative to the other quadrature polarization component is delayed by π/2, and then the original linearly polarized beam becomes an elliptically polarized beam. The elliptically and linearly polarized beams reflected by the retroreflectors overlap in space, causing interference. When the interference light passes through the polarizing beam splitter (PBS) (Thorlabs, Newton, NJ, USA, PBS10-1550), the s-polarized light is reflected, and the p-polarized light is transmitted and then refracted by a right-angle prism mirror (Thorlabs, Newton, NJ, USA, MRA10-P01). The two beams of light are received by the two couplers (Oz optics, Carp, ON, Canada, HPUCO-23AF-1300/1550-S-2.7AS) into the fiber and obtained by the PD (Shanghai Aoxiu Information Technology, Shanghai, China, PDA1005-8-B). A retroreflector placed in the optical arm of the HQLI with the OWP is installed on a one-dimensional displacement stage with a micrometer (Zolix, Beijing, China, KSM25A-65C), which has a minimum scale of 0.01 mm, thereby adjusting the OPD.

## 4. Results and Discussions

### 4.1. Real-Time OPD Measurement

**Figure 4:**
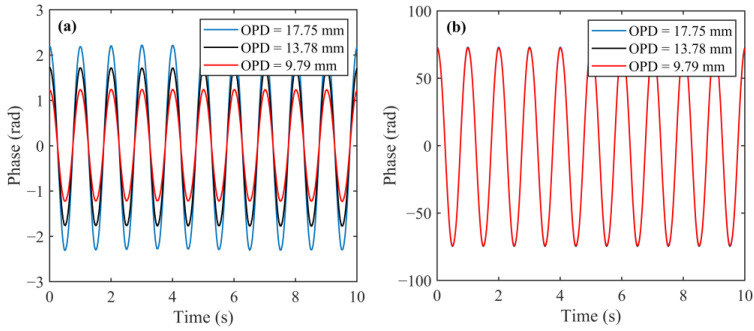
Phase change of HQLI (**a**) and phase change of auxiliary 3 × 3 interferometer (**b**) under different OPDs of HQLI.

NKT Photonics software is used to perform sinusoidal wavelength modulation on the E15 laser. The wavelength modulation frequency is set to 1 Hz, and the wavelength modulation amplitude is set to an appropriate value. The micrometer is rotated to change the position of the retroreflector (namely, the OPD of the HQLI). The phase changes of the HQLI and auxiliary 3 × 3 interferometer corresponding to each position are used to calculate the OPD of the HQLI. Each measurement is averaged over ten acquisitions. As shown in Figure 4, the retroreflector of the HQLI is adjusted to three different positions, and the OPD of the HQLI at the three positions is calculated as 17.75 mm, 13.78 mm, and 9.79 mm, respectively. Under different OPDs, the phase change amplitude of the auxiliary interferometer is unchanged. The phase change amplitude of the HQLI decreases as its OPD decreases. The phase changes are demodulated in real time, so the OPD of the HQLI can be measured in real time.

**Figure 5:**
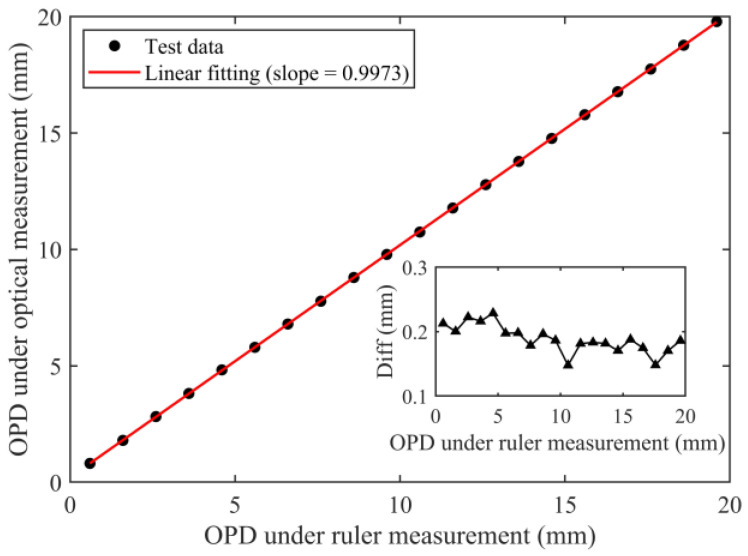
Measurement results of OPD.

When the position of the retroreflector is adjusted, the OPD of the HQLI is measured by Equation (5), which is compared with the result of the ruler measurement, as shown in Figure 5. Figure 5 shows the OPD measurement range of 0~20 mm. The optical measurement result is linearly related to the ruler measurement result, with a slope of approximately 1. The illustration shows the difference between the two results. When the OPD is greater than 7 mm, the average difference between the two is 0.17 mm, which can be used to correct the ruler measurement results. As the OPD gradually decreases, the average value of the difference is 0.21 mm. The cause is that the measurement error of the optical phase change of the HQLI increases when the OPD is small.

**Figure 6:**
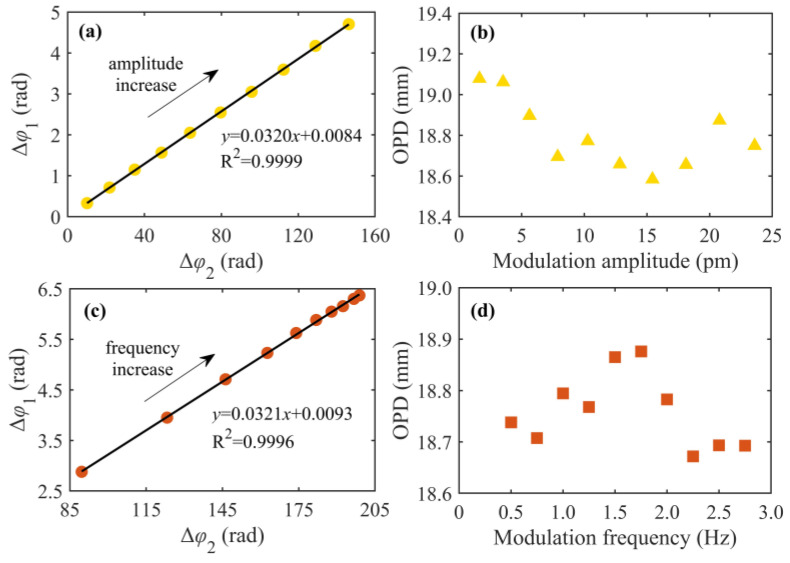
Phase changes (**a**,**c**) and OPD results (**b**,**d**) under different modulation amplitudes and frequencies.

To demonstrate the influence of different wavelength modulation amplitudes and frequencies on real-time OPD measurements, the amplitude and frequency of sinusoidal wavelength modulation are changed when the micrometer is fixed at a scale of 25 mm. Figure 6a,b demonstrate the results at different modulation amplitudes with a modulation frequency of 1 Hz. The phase changes of the two interferometers increase linearly with increasing modulation amplitude. The average value of the OPD is 18.74 mm when the modulation amplitude is above 5 pm. When the modulation amplitude is less than 5 pm, the average OPD is 19.07 mm and has a large measurement error. Because the wavelength modulation amplitude is small, the phase change peak-to-peak value of the HQLI is less than π/2, which makes the fitted ellipse parameters unable to compensate for the light-intensity fluctuation in real time, resulting in large measurement errors in the phase change and OPD result.

When the wavelength modulation frequency changes, the phase changes, and the OPD results are shown in Figure 6c,d. Because the modulated response of the laser varies in a specific frequency range, the increase in the modulation frequency increases the amplitude, and the phase change of the two interferometers linearly increases. At a modulation frequency of 0.5~2.75 Hz, the average value of the OPD is 18.76 mm, close to the value of 18.74 mm measured under different amplitudes.

In summary, through real-time phase comparison between the HQLI and the auxiliary 3 × 3 interferometer, the OPD of the HQLI can be measured. Different modulation amplitudes and frequencies have no significant influence on real-time OPD measurements. Given the EFA based on the Kalman filter used, the product of the wavelength modulation amplitude and the OPD should be guaranteed to be greater than or equal to *λ*^2^/16 to minimize the phase demodulation errors.

### 4.2. Uncertainty Evaluation Analysis

Based on the OPD measurement results, the OPD measurement uncertainty of the HQLI needs to be analyzed in detail. The values of various physical quantities mentioned below are the arithmetic average values obtained from repeated measurements.

The OPD measurement uncertainty of the HQLI is related to the phase change of the HQLI, the phase change of the auxiliary 3 × 3 interferometer, and the interference spectrum of the auxiliary 3 × 3 interferometer. The standard deviation of the arithmetic mean is calculated as *σ_λ_*_1_ = 46.31 fm for *λ*_1_ and *σ_λ_*_2_ = 46.61 fm for *λ*_2_. The resolution of the OSA is 0.04 pm, and its corresponding uncertainty *u_r_* is 0.02 pm. The phase demodulation accuracy is taken as *σ_p_* = 0.001 rad. In addition, the standard deviations *σ*_Δ*φ*1_ and *σ*_Δ*φ*2_ of the phase changes of each OPD in ten repeated measurements are used as the uncertainties.

**Figure 7:**
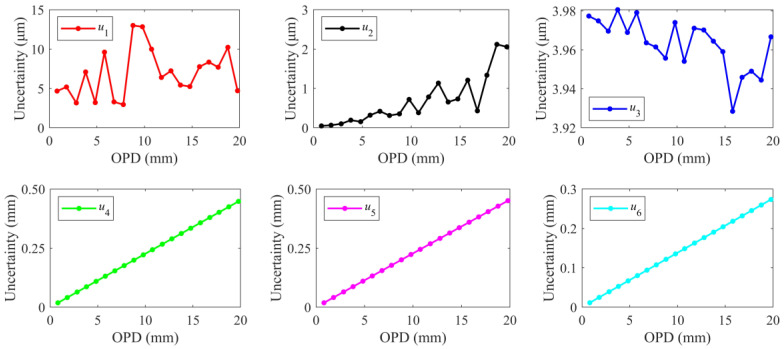
Uncertainty components under different OPDs.

The standard uncertainty components *u*_1_, *u*_2_, *u*_3_, *u*_4_, *u*_5_, and *u*_6_ are shown in Figure 7. The components *u*_4_, *u*_5_, and *u*_6_ caused by OSA scanning are linearly related to the OPD of the HQLI, respectively, which is mainly determined by the ratio of Δ*φ*_1_/Δ*φ*_2_. The component *u*_2_ caused by Δ*φ*_2_ tends to increase as the OPD increases. Ideally, the calibrated OPD of the auxiliary 3 × 3 interferometer remains unchanged, so regardless of the OPD of the HQLI, the arithmetic mean of Δ*φ*_2_ and the corresponding standard deviation are the same. The component *u*_2_ related to Δ*φ*_1_ increases with the OPD of the HQLI. However, the frequency fluctuation of the laser causes the arithmetic mean and standard deviation of Δ*φ*_2_ calculated at each OPD to be inconsistent, causing *u*_2_ to fluctuate. *U*_1_ is caused by the measurement repeatability of Δ*φ*_1_. Since Δ*φ*_1_ is inconsistent under different OPDs, the corresponding standard deviation σ_Δ*φ*1_ is greatly affected by random errors, so there is no obvious rule. *U*_3_, caused by phase demodulation accuracy, theoretically increases with the OPD of the HQLI. However, due to the frequency fluctuation of the laser, the arithmetic mean of Δ*φ*_2_ calculated at each OPD is slightly different, resulting in *u*_3_ being random.

**Figure 8:**
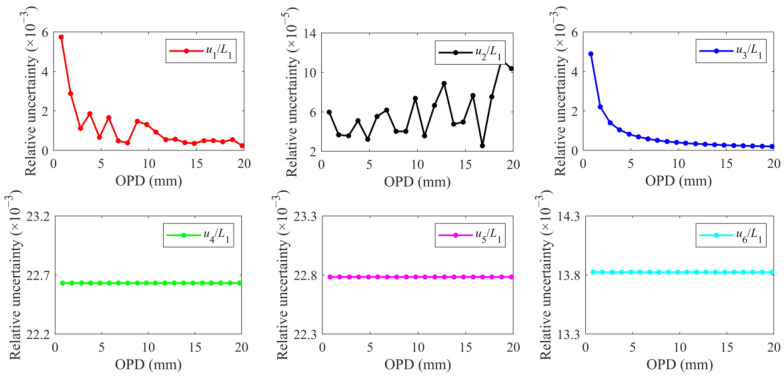
Relative uncertainty components under different OPDs.

Each relative uncertainty component is calculated and is shown in Figure 8. The relative uncertainty components *u*_4_/*L*_1_, *u*_5_/*L*_1_, and *u*_6_/*L*_1_ majorly contribute to the total relative uncertainty and are consistent under different OPDs. Their values are one to two orders of magnitude greater than those of the other three components. The component *u*_2_/*L*_1_ of the measurement repeatability of Δ*φ*_2_ is almost negligible, but it reflects the random error introduced by repeated measurements. The components *u*_1_/*L*_1_ and *u*_3_/*L*_1_ corresponding to the measurement repeatability and phase demodulation accuracy of Δ*φ*_1_, respectively, are mainly determined by the measured value of Δ*φ*_1_. As the OPD increases, Δ*φ*_1_ increases, so *u*_1_/*L*_1_ and *u*_3_/*L*_1_ decrease. *u*_1_/*L*_1_ and *u*_3_/*L*_1_ also determine that the total relative uncertainty decreases as the OPD increases, proving the advantages of this scheme for long-distance ranging. Therefore, for the real-time OPD measurement of the main interferometer, the OSA scanning that calibrates the 3 × 3 interferometer is the main contribution to the OPD measurement uncertainty of the HQLI.

### 4.3. Uncertainty Comparison under Different Calibration Methods of the Auxiliary Interferometer

Furthermore, another method is used to calibrate the auxiliary interferometer, which aims to analyze the impact of the auxiliary interferometer on the OPD measurement of the main interferometer under different calibration methods. Here, ruler measurement is a relatively simple method to use. The arm length difference of the auxiliary 3 × 3 interferometer is measured with a steel ruler, and the OPD is calculated based on the refractive index of the fiber core. Then, the main interferometer’s OPD measurement formula is

(14) $$L_{1}=\frac{\Delta \varphi_{1}}{\Delta \varphi_{2}}\cdot n\left(l_{l}-l_{s}\right),$$

where *l_l_* and *l_s_* are the lengths of the two fiber arms of the auxiliary 3 × 3 interferometer, and *n* is the refractive index of the fiber core, which is taken as a constant value of 1.4682. For ruler measurements, the OPD measurement uncertainty of the HQLI mainly considers the uncertainty components contributed by the measurement repeatability of Δ*φ*_1_, Δ*φ*_2_, *l_l_*, and *l_s_*, the phase demodulation accuracy, and the resolution of the steel ruler, corresponding to $u_{1}^{\prime }$, $u_{2}^{\prime }$, $u_{4}^{\prime }$, $u_{5}^{\prime }$, $u_{3}^{\prime }$, and $u_{6}^{\prime }$, respectively. The uncertainty and relative uncertainty when measuring the main interferometer’s OPD are evaluated as

(15) $$\begin{array}{ll} u^{\prime } & =\sqrt{{u_{1}^{\prime }}^{2}+{u_{2}^{\prime }}^{2}+{u_{3}^{\prime }}^{2}+{u_{4}^{\prime }}^{2}+{u_{5}^{\prime }}^{2}+{u_{6}^{\prime }}^{2}}\\ & =\sqrt{\begin{array}{l} {\left[\frac{1}{\Delta \varphi_{2}}\cdot n\left(l_{l}-l_{s}\right)\right]}^{2}\sigma_{\Delta \varphi_{1}}^{2}+{\left[-\frac{\Delta \varphi_{1}}{\Delta {\varphi_{2}}^{2}}\cdot n\left(l_{l}-l_{s}\right)\right]}^{2}\sigma_{\Delta \varphi_{2}}^{2}+\left\{{\left[\frac{1}{\Delta \varphi_{2}}\cdot n\left(l_{l}-l_{s}\right)\right]}^{2}+{\left[-\frac{\Delta \varphi_{1}}{\Delta {\varphi_{2}}^{2}}\cdot n\left(l_{l}-l_{s}\right)\right]}^{2}\right\}\sigma_{p}^{2}\\ +{\left(\frac{\Delta \varphi_{1}}{\Delta \varphi_{2}}\cdot n\right)}^{2}\sigma_{l_{l}}^{2}+{\left(-\frac{\Delta \varphi_{1}}{\Delta \varphi_{2}}\cdot n\right)}^{2}\sigma_{l_{s}}^{2}+\left[{\left(\frac{\Delta \varphi_{1}}{\Delta \varphi_{2}}\cdot n\right)}^{2}+{\left(-\frac{\Delta \varphi_{1}}{\Delta \varphi_{2}}\cdot n\right)}^{2}\right]{u_{r}^{\prime }}^{2} \end{array}} \end{array},$$

(16) $$\begin{array}{ll} \frac{u^{\prime }}{L_{1}} & =\sqrt{{\left(\frac{u_{1}^{\prime }}{L_{1}}\right)}^{2}+{\left(\frac{u_{2}^{\prime }}{L_{1}}\right)}^{2}+{\left(\frac{u_{3}^{\prime }}{L_{1}}\right)}^{2}+{\left(\frac{u_{4}^{\prime }}{L_{1}}\right)}^{2}+{\left(\frac{u_{5}^{\prime }}{L_{1}}\right)}^{2}+{\left(\frac{u_{6}^{\prime }}{L_{1}}\right)}^{2}}\\ & =\sqrt{{\left(\frac{\sigma_{\Delta \varphi_{1}}}{\Delta \varphi_{1}}\right)}^{2}+{\left(\frac{\sigma_{\Delta \varphi_{2}}}{\Delta \varphi_{2}}\right)}^{2}+\left[{\left(\frac{1}{\Delta \varphi_{1}}\right)}^{2}+{\left(\frac{1}{\Delta \varphi_{2}}\right)}^{2}\right]\sigma_{p}^{2}+{\left(\frac{\sigma_{l_{l}}}{l_{l}-l_{s}}\right)}^{2}+{\left(\frac{\sigma_{l_{s}}}{l_{l}-l_{s}}\right)}^{2}+2{\left(\frac{u_{r}^{\prime }}{l_{l}-l_{s}}\right)}^{2}} \end{array},$$

where *σ_ll_* is the standard deviation of the average value of repeated measurements of *l_l_*; *σ_ls_* is the standard deviation of the average value of repeated measurements of *l_s_*; and $u_{r}^{\prime }$ is the uncertainty corresponding to the resolution *r*′ of the steel plate ruler, taking a uniform distribution, $u_{r}^{\prime }$ = *r*′/√3.

The arm length of the auxiliary 3 × 3 interferometer is measured by using a steel ruler with a resolution of 1 mm. Ten repeated measurements are performed, and the average values are obtained. Therefore, *l_l_* = 803.52 mm, *σ_ll_* = 0.03887 mm, *l_s_* = 405.05 mm, and *σ_ls_* = 0.01667 mm. Using the above phase comparison results, we can recalculate the OPD of the HQLI and its measurement uncertainty by using Equations (15) and (16).

**Figure 9:**
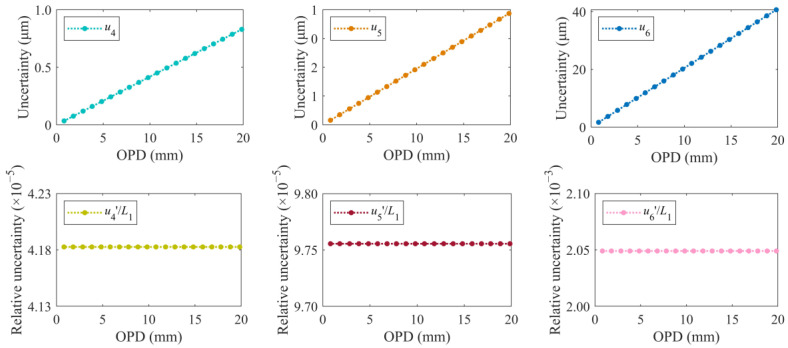
Uncertainty and relative uncertainty components under different OPDs caused by the ruler measurement.

It is evident that no matter what calibration method is used for the auxiliary interferometer, as long as the OPD results are proximate, the uncertainty components corresponding to the phase comparison are almost unchanged. Thus, Figure 9 only shows the uncertainty and relative uncertainty components caused by ruler measurements. When calibrating the auxiliary 3 × 3 interferometer, the ruler measurement causes less OPD uncertainty than OSA scanning. The trough measurement and OSA performance limit OSA scanning, and this causes a relative uncertainty limited to more than 10^−2^. The relative uncertainty introduced by ruler measurements for the calibration of the auxiliary interferometer is reduced by one to two orders of magnitude compared with that corresponding to the abovementioned OSA scanning, equivalent to the relative uncertainty introduced by phase comparison.

**Figure 10:**
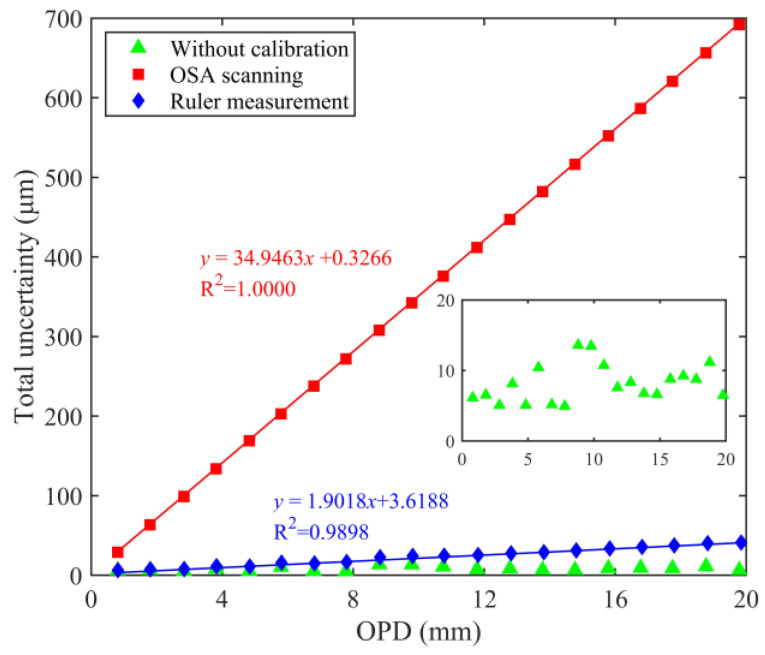
Relationship between total uncertainty and OPD.

**Figure 11:**
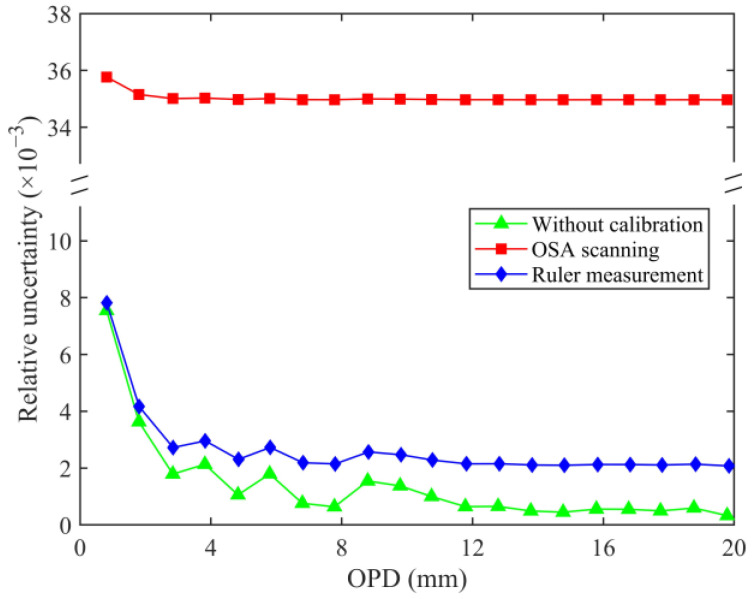
Comparison of three relative uncertainties.

Figure 10 shows the total uncertainty for the three cases, and Figure 11 shows the corresponding total relative uncertainty. Whether it is OSA scanning or ruler measurements, the corresponding auxiliary 3 × 3 interferometer OPD calibration contributes majorly to the total uncertainty. The two methods also determine the linear relationship between the OPD and the total uncertainty. Within a specific measurement range, the OPD measurement uncertainty of the HQLI can be predicted. Regardless of the influence of the auxiliary interferometer calibration, the total uncertainty represented by the green symbol is only the synthesis of *u*_1_, *u*_2_, and *u*_3_ (or $u_{1}^{\prime }$, $u_{2}^{\prime }$, and $u_{3}^{\prime }$), corresponding to the phase comparison. A random relationship exists between the OPD and the total uncertainty, as shown in the illustration in Figure 10. Moreover, it can still be found that the relative uncertainty component of the phase comparison determines the trend and fluctuation in the total relative uncertainty. The total uncertainty corresponding to the ruler measurement is smaller than that of OSA scanning.

Here, OSA scanning is used to calibrate the auxiliary 3 × 3 interferometer instead of using a ruler with smaller measurement uncertainty because of the nonuniformity of the refractive index of the optical fiber. The OPD of the auxiliary 3 × 3 interferometer calibrated by OSA scanning is more credible. However, this approach does not prevent us from analyzing the impact of ruler measurements on the OPD measurement of the main interferometer. In any case, the calibration of the auxiliary interferometer significantly contributes to the OPD measurement uncertainty of the main interferometer. Choosing an accurate calibration method can reduce the OPD measurement uncertainty to close to the uncertainty limit contributed by the phase comparison.

For phase-generated carrier-modulated interferometric sensors with arm length differences at the ~m level, such as geophone arrays requiring strict control of arm length differences to reduce the inconsistency of the carrier modulation depth [28] and accelerometers requiring optimal arm length differences to obtain low phase noise [10], this measurement scheme can ensure the best uncertainty of 1 mm. In addition, the proposed scheme and uncertainty evaluation can also be used for real-time OPD adjustment of a laser interferometer for gravitational wave detection [29], reducing the mm level OPD to below mm, with an error of tens of μm, which is close to the uncertainty limit introduced by phase comparison.

Some OPD measurement schemes mentioned in the introduction were initially developed for absolute ranging. The scheme that we are using now can also be used for ranging. Its measurement range depends on the phase demodulation algorithm and the wavelength tuning amplitude of the laser. It is well known that the upper limit of the demodulation phase of the EFA can reach thousands of rad at a small modulation frequency (<10 Hz). Considering the wavelength modulation amplitude of 1 pm, the measurement range of the proposed scheme can reach hundreds of meters or even kilometers. As for the measurement accuracy, increasing the OPD of the auxiliary interferometer can achieve a smaller OPD measurement uncertainty of the main interferometer. With the support of a large OPD auxiliary interferometer, the relative uncertainty is expected to decrease as the OPD of the main interferometer increases, confirming the advantage of long-distance ranging. However, the stability of the auxiliary interferometer is undoubtedly a complex problem. Here, only the temperature, sound, and vibration isolation package are used to keep the OPD of the auxiliary interferometer stable during short-term measurements. It is necessary to calibrate the auxiliary interferometer before each use.

## 5. Conclusions

Real-time OPD measurement of a main interferometer through laser wavelength modulation and an auxiliary interferometer is demonstrated, and the OPD measurement uncertainty is analyzed. Different modulation amplitudes and frequencies do not affect the real-time feasibility of the OPD measurement. The sources of the OPD measurement uncertainty are phase comparison and auxiliary interferometer calibration. The auxiliary interferometer calibration method contributed considerably to the OPD measurement uncertainty in the experiment. As the OPD increases, the phase comparison determines the trend and fluctuation in the relative uncertainty, while the auxiliary interferometer has a constant value in the relative uncertainty. Moreover, the relative uncertainty of the OPD decreases as the OPD increases, showing the advantage of long-distance ranging. Our uncertainty evaluation proves that high-precision main interferometer OPD measurements can only be achieved by selecting a suitable calibration method for auxiliary interferometers. In addition, enlarging the calibrated OPD of the auxiliary interferometer and ensuring its stability are also crucial to substantially improving the measurement accuracy. In terms of applications, the proposed scheme and uncertainty evaluation can be used for vibration, hydroacoustic, and magnetic field measurements with OPDs of the ~m level to ensure that the measurement uncertainty can be close to the limitation of phase comparison, which is the better method for reducing the system noise and improving the system’s consistency.

## Data Availability

The data are not publicly available due to the confidentiality and nondisclosure agreement with the funders.
